# Brand Discrimination: An Implicit Measure of the Strength of Mental Brand Representations

**DOI:** 10.1371/journal.pone.0121373

**Published:** 2015-03-24

**Authors:** Mike Friedman, Thomas Leclercq

**Affiliations:** Center for Research on Consumers and Marketing Strategy (CCMS), Louvain School of Management, Catholic University of Louvain, Mons, Belgium; Universitat de Valencia, SPAIN

## Abstract

While mental associations between a brand and its marketing elements are an important part of brand equity, previous research has yet to provide a sound methodology to measure the strength of these links. The following studies present the development and validation of an implicit measure to assess the strength of mental representations of brand elements in the mind of the consumer. The measure described in this paper, which we call the Brand Discrimination task, requires participants to identify whether images of brand elements (e.g. color, logo, packaging) belong to a target brand or not. Signal detection theory (SDT) is used to calculate a Brand Discrimination index which gives a measure of overall recognition accuracy for a brand’s elements in the context of its competitors. A series of five studies shows that the Brand Discrimination task can discriminate between strong and weak brands, increases when mental representations of brands are experimentally strengthened, is relatively stable across time, and can predict brand choice, independently and while controlling for other explicit and implicit brand evaluation measures. Together, these studies provide unique evidence for the importance of mental brand representations in marketing and consumer behavior, along with a research methodology to measure this important consumer-based brand attribute.

## Introduction

Creating and maintaining strong brands is an enormous priority for today’s marketers and organizations. Companies invest a great deal of time, money and effort in building and maintaining coherent brands, in which branding elements (e.g. slogans, colors, packaging) are all coherent with one another, strongly linked to the parent brand, and unique from competitor brands in the same category. Furthermore, a great deal of marketing budgets is spent on strengthening links among brand elements in the minds of consumers through marketing communication and advertising. Therefore, understanding the uniqueness and strength of consumers’ mental representation of brands and their elements is vital from a marketing perspective.

However, this area remains under-studied in the existing marketing and consumer behavior literature. A great deal of research attention has been given to understanding explicitly-measured, reflective brand constructs such as attitudes [1], emotions [2] and personality [3], as well as the inter-relation among them [4]. While these areas of research have contributed much to current knowledge, they neglect a critical dimension of consumer brand understanding: the uniqueness and strength of brands’ elements in consumers’ memory, in comparison with close competitors. A number of qualitative and questionnaire-based approaches have been developed to understand mental models of brands, yet these techniques remain ill-suited to measure the strength of connections of brand elements in consumer memory with precision. This is indeed a critical issue in that consumers’ mental representations of brands and their elements are thought to constitute a key aspect of consumer-based brand equity [5,6].

The goal and contribution of the current research is to develop and validate an implicit methodology which can be used to assess consumers’ mental representation of brands. In this technique, participants are exposed to a series of brand elements (e.g. logo, color, packaging shape) and must decide, within a fixed response deadline, whether each image belongs to a target brand or not. The task presents both images belonging to the target brand and images that do not belong to the target brand (e.g. images of competing brands’ elements). Signal detection theory (SDT) is used to calculate a Brand Discrimination index which gives a measure of overall recognition accuracy for a brand’s elements in the context of its competitors.

The research presented in this article describes and demonstrates the validity of the Brand Discrimination exercise, using a variety of brands in a number of different sectors. We show that mental representations of a brand and its elements form an important part of consumer-based brand equity, and that these representations can predict brand choice, even when controlling for other explicit and implicit brand constructs.

## Theoretical Development

### Traditional Measurement of Consumer Perceptions of Brands

According to the American Marketing Association, a brand is a “name, term, design, symbol, or any other feature that identifies one seller's good or service as distinct from those of other sellers” (AMA). Much consumer research has focused on understanding consumers’ reflective evaluations, attitudes, and feelings about brands. This type of research uses explicit questionnaire measures to understand constructs such as brand attitude [7,8] and emotions [2], along with other reflective perceptions such as perceived masculinity and femininity [9], brand experience [10], and brand attachment [11]. This work has greatly expanded scholars’ understanding of the broader nomological networks of the different types of attitudes, beliefs, and perceptions that consumers can have about brands. However, these reflective explicit measures are not particularly well-suited to understanding the uniqueness and strength of consumers’ mental representations of brands and their marketing elements.

### Brands as Associative Networks

Associative network theories of cognition [12] provide a useful theoretical framework for understanding the strength of brand associations in consumer memory. According to the associative network perspective, brands are represented in consumers’ memory as networks of mental representations that are linked by connections that vary in strength [6]. At this fundamental level of representation, brands exist as cognitive structures which consist of clusters of meanings and associations, with many different elements organized in network-like arrangements [4,6,13–15].

From a marketing point of view, it is critical to understand the nature of these mental representations of brands in consumer memory [4]. Of particular importance from a branding perspective are the strength and uniqueness of a brand’s associations in relation to its competitors. Because a primary objective of brands is to differentiate a seller’s product from those of its competitors, creating and maintaining strong brand-element associations which are unique relative to other brands in the category are often primary marketing objectives [6,13]. Indeed, the uniqueness and strength of a brand’s associations in relation to its competitors is widely-viewed as a key component of consumer-based brand equity [4–6,16–18].

From the associative network perspective, greater co-occurrence between a brand and its elements (such as its logo or colors) should strengthen these mental representations and the links between them [19,20]. This repeated exposure can occur in at least two ways. First, direct experience with the brand is thought to help strengthen brand associations [6,13], for example when users of a brand are exposed repeatedly to the brand and its elements in conjunction with one another (e.g. the name and logo of a given brand on a box of cereal). Second, repeated exposure to a brand and its elements through marketing communications should help create strong and unique brand associations, and strengthen existing associations [6,13,19]. Indeed, a major goal of certain types of marketing actions is to create or strengthen brand associations [6].

Furthermore, the strength and uniqueness of consumers’ mental representation of brands should be predictive of brand choice [6]. First, choice is a function of information represented in memory [19], and strong, easily-accessible mental representations are thought to help guide the choice process in some circumstances [21]. Indeed, the notion of category and instance dominance (e.g. the advantage inherent to the most-easily recalled brand for a category or product choice [22]) suggests that the strength of mental associations determines whether people consider brands, which then influences subsequent brand choices [19,23]. Second, scholars have suggested that brand recognition or familiarity (facilitated by strong mental representations of a brand) can serve as a choice heuristic, with high familiarity serving as a diagnostic cue to impact brand choice [24].

Current measurement approaches to understanding the strength and uniqueness of branding element associations have some notable limitations. Most existing methods are qualitative or questionnaire-based, and seek to elicit qualities or traits associated with brands [5,15,25]. However, these types of measures, requiring conscious reflection on the part of consumers, represent approximations at best of the strength and uniqueness of mental representations of brands and their branding elements [19]. There is therefore a need for a sophisticated and rigorous methodology to measure the uniqueness and strength of a brand’s mental representation, in comparison with close competitors. Such a measure can potentially serve as an important consumer-based marketing metric in its own right. In the current paper, we fill this gap in the literature by describing and validating a speeded reaction time categorization task to assess the uniqueness and strength of mental representations of brands and their elements versus those of close competitors.

### Implicit Measurement and the Brand Discrimination Task

#### Implicit measurement

We characterize the measure developed in this paper as implicit. Many different definitions of this term have been proposed, and the word “implicit” has been used somewhat ambiguously to refer to both mental processes and to measurement techniques (see [26–28] for detailed discussions of this issue). In the work presented in this article, we employ the operational definition proposed by Gawronski [29], who defines implicit measures as research techniques that “infer mental contents from participants’ performance on experimental paradigms, most often speeded categorization tasks” (pp. 141). Thus, the methodology we develop in this paper is implicit in that it seeks to infer mental contents (consumer representations of brands and their elements versus the competition) from participants’ performance on an experimental task (a speeded identification task of brand elements). Other types of implicit measures, such as the Implicit Association Test, have been fruitfully used in marketing and consumer behavior research (see [30,31] for recent reviews)

#### The Brand Discrimination Task

The Brand Discrimination task consists of a series of trials, presented via computer. In each trial, a target brand is displayed at the top of the screen (e.g. Kit Kat). After 500 ms, a brand image (e.g. a picture of a Kit Kat package) is shown in the center of the computer screen for a given period of time (the response deadline). Participants must decide whether the brand image displayed in the center of the screen matches the target brand displayed at the top of the screen. If the image matches the target brand, participants must respond by hitting the space bar. If the image does not match the target brand, the participants must do nothing (e.g. not hit the space bar). A 500 ms interstimulus interval (ISI) occurs the end of each trial, determined either by the participant’s response or the end of the response deadline (whichever comes first); during the ISI the brand stimuli are removed from the screen. In keeping with other validated implicit measures (e.g. the IAT [32], AMP [33], and GNAT [34]), the ISI values for the Brand Discrimination Task are constant across trials. The underlying logic is that the constancy of the ISI across trials allows for less variation in responses across trials.

The brand discrimination task consists of two types of trials. In *target present trials*, the brand elements shown on the screen are related to the target brand (e.g. a Kit Kat image is shown on the screen when the target brand is Kit Kat). In *target absent trials*, the brand elements shown on the screen belong to a competing brand (e.g. a Snickers or Twix image is shown on the screen when the target brand is Kit Kat). The number of trials in a given exercise is based on the number of brand stimuli in that particular exercise (see [33] for a conceptually similar approach). Specifically, each image related to a target brand (e.g. each branding element chosen for Kit Kat) is shown three times in target present trials (e.g. when the target brand is Kit Kat). In target absent trials, non-target brand stimuli are taken from competing brands in the product category.

For each target brand under study, the exercise consists of an equal number of target present and target absent trials, with trial order randomly determined for each participant. Therefore, although the constant ISI means that participants can anticipate *when* a given stimulus will appear (and thus pay a maximum amount of attention), randomization of stimulus presentation across trials ensures that participants cannot anticipate *what* stimulus will appear in a given trial. In sum, there is no efficient guessing strategy because the overall probability that a target stimulus will be shown in a given trial is exactly 50%. In all cases, the main Brand Discrimination exercise is preceded by 8 practice trials, in order to familiarize the participants with the task. In the practice trials, the brands Coca-Cola and Pepsi each serve as the target brand for 4 trials.

Signal Detection Theory (SDT; [35]) is used to calculate a Brand Discrimination score for each brand in an exercise. This Brand Discrimination score is quantified by the SDT measure d’ (d prime), which takes into account both correct and incorrect responses on the recognition task. Formally, this index is calculated as the probit of the proportion of correct responses on target present trials (“hits” in the SDT framework) minus the probit of the proportion of incorrect responses on target absent trials (“false alarms” in the SDT framework). Corrections are applied to cells with perfect hit and zero error rates (for details see [36]). For a review of signal detection theory see [35–37].

### Summary and Research Overview

We argue that the mental associations between a brand and its elements are an important part of brand strength, but that previous research has yet to provide a sound methodology to measure these links. The following studies present the development and validation of an implicit measure to assess mental representations of brand elements in the mind of the consumer. The measure described in this paper, which we call the Brand Discrimination task, requires participants to identify whether images of brand elements (e.g. color, logo, packaging) belong to a target brand or not.

Study 1 A provides evidence for known-groups validity of the task by showing that participants are better able to discriminate the elements of a Market Leading brand, as compared to two weaker competitors. Study 1 B investigates consumer recognition of these same brands, and shows that (consistent with associative network theories of branding), when the response deadline is shortened, only users of the Market Leader brand are able to discriminate between that brand and its two weaker competitors. Study 2 provides experimental evidence for the validity of the Brand Discrimination measure. Specifically, in Study 2 we manipulate the underlying attribute the task measures (the strength of mental associations between a brand and its elements), and demonstrate that Brand Discrimination scores vary as a function of this manipulation. This approach has been explicitly advocated as a way to demonstrate the validity of implicit measures [29,38]. Study 3 demonstrates evidence for the temporal stability of the Brand Discrimination measure by showing acceptable test-retest reliability at a 3 week time interval. Study 4 shows that the Brand Discrimination measure is predictive of consumer choice, even when controlling for explicit brand constructs (brand attitude and affect). Study 5 provides a replication of this finding and shows that Brand Discrimination predicts consumer choice when controlling for explicit brand constructs and implicit brand attitudes.

## Study 1 A

The purpose of Study 1 A was to provide evidence for the known-groups validity of the Brand Discrimination exercise. To this end, we employed the procedure to study three consumer food brands in a large European country, in collaboration with a multi-national fast-moving consumer goods (FMCG) company. One of the brands was the Market Leader brand (based on market share and distribution) in the country under study. The other two brands were weaker competitors in the category. (Due to confidentiality reasons, the names of the brands have been anonymized.)

In Study 1 A we hypothesize that participants should be better able to discriminate the Market Leader (ML) brand, as compared to two competitor brands. As an exploratory analysis, we examined whether the pattern of Brand Discrimination scores was different for users and non-users of the Market Leader brand. According to associative network theories of branding, one might expect that users of the ML brand would better at discriminating among the brands. However, this difference might only be visible when brand elements must be recognized quickly, e.g. at shorter response deadlines. Indeed, it is precisely under such circumstances that strong mental connections created by brand use should facilitate Brand Discrimination.

### Pre-test

Image selection was done in collaboration with brand managers at the Market Leader company. The brand visuals were defined in terms of the important marketing elements for the category: packaging visuals, pack shapes (silhouettes), logos, colors, slogans, advertising images, and brand themes (e.g. visual images evocative of the brand architecture). For each of the three brands, visual stimuli corresponding to each of these categories were chosen, resulting in 21 total stimuli (7 per brand).

In order to ensure that the images chosen by the brand managers and the authors of this paper were indeed reflective of their respective brands, we pre-tested all the images used in this study. Specifically, participants (*N* = 15) evaluated all images on 7-point scales (from *not at all* to *very much*), rating the extent to which each image was related to each of the three brands under study. Results revealed that participants rated the images as belonging more to the appropriate brand than for the other two, F’s for the Market Leader, Competitor 1 and Competitor 2 brands of *F* (2, 28) = 122.98, *p* < .001, *F* (2, 28) = 73.82, *p* < .001, *F* (2, 28) = 164.63, *p* < .001, respectively.

### Method

Participants were 96 members of an online market research panel (27 men, mean age = 41.36 *SD* = 12.79) who participated in return for the chance to win a prize of 300 euros in value. Informed consent was presented in written form at the beginning of the survey. Participants were informed that they would participate in a research study about marketing stimuli, and clicked a radio button indicating their agreement to do so. Only after indicating their agreement were participants allowed to continue to the study. The publicly posted data from this study have been anonymized.

Participants first completed a series of Brand Discrimination exercises, which were presented in three blocks of trials. In each block, a single brand served as the target brand. Each block contained a total of 21 target present trials (the 7 target brand elements shown 3 times each) and 21 target absent trials (with brand elements randomly chosen from the non-target brands). Block order was counter-balanced across participants. The response deadline for responding to each stimulus was 1200 ms. Internal consistency for the exercises for the Market Leader brand, Competitor 1 and Competitor 2, were .90, .88 and .88, respectively. At the end of the study, participants indicated their age, gender, and whether they were current users of the brand (with at least one usage occasion within the past 3 months). We explored the effects of gender (e.g. main effects and interactions) in the data for Study 1 A as well as for all subsequent studies. Because no statistically significant sex-of-participant effects emerged in these data, this variable will not be discussed further.

### Ethics Statement

In Belgium there is no legal requirement to obtain approval from an institutional review board (IRB) for non-clinical research studies. The authors work in a university in which no IRB existed at the time when the data were collected.

### Results

#### Brand Discrimination Scores

Our primary hypothesis was that Brand Discrimination would be higher for the Market Leader brand, as compared to the two competing brands.

**Fig 1:**
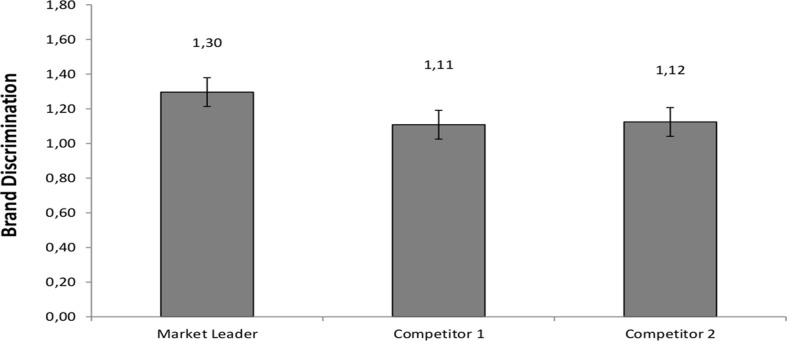
Brand Discrimination for the Market Leader brand and its two competitors, Study 1 A. Note: Standard error bars are shown for each mean.

This hypothesis was tested in a repeated-measures ANOVA, with Brand Discrimination (Market Leader vs. Competitor 1 vs. Competitor 2) as a within-subjects factor. This analysis indicated a statistically significant within-subject effect, *F* (2, 190) = 3.57, *p* = .03, *ηρ²* = .04, see Fig. 1. We decomposed this effect by conducting a contrast analysis. Specifically, in keeping with our knowledge of the relative positions of the brands in the marketplace, we compared the Brand Discrimination score for the Market Leader brand versus the average Brand Discrimination of the two competing brands. The contrast analysis was statistically significant *F* (1, 95) = 6.95, *p* = .01, *ηρ²* = .07. (The complementary orthogonal contrast compared Brand Discrimination scores between Competitor 1 and Competitor 2 brands, and was not statistically significant, *F* (1, 95) = .05, *p* = .83).

#### Moderation of Brand Discrimination by Brand Use

To determine whether the observed pattern of Brand Discrimination was identical for both users and non-users of the Market Leading brand, Brand Discrimination scores were submitted to a 3 (Brand Discrimination: Market Leader vs. Competitor 1 vs. Competitor 2) X 2 (Market Leader Brand use: yes vs. no) mixed model ANOVA, with repeated-measures on the first factor. In this analysis, the Brand Discrimination by ML Brand Use interaction was not statistically significant, *F* (2, 188) = .51, *p* = .60, indicating that the pattern of within-subjects Brand Discrimination scores reported above did not differ between users and non-users of the Market Leading Brand.

### Discussion

The findings from Study 1 A show that participants had higher Brand Discrimination scores for the Market Leader brand as compared to its two weaker competitors, providing evidence for the known-groups validity of the Brand Discrimination measure as an index of mental representation of brand elements. Furthermore, at the response deadline of 1200 ms, there were no differences in brand discrimination between users and non-users of the Market Leader brand.

From a theoretical perspective, use of a given brand (and therefore exposure to and reinforcement of its brand elements) should strengthen mental representations of that brand. One might therefore expect that, contrary to the findings of Study 1 A, users of a brand should be better able to discriminate that brand from its competitors. However, it is possible that this pattern might only be observable when identification of brand elements must be done quickly, e.g. at shorter response deadlines than that used in Study 1 A. We explored this possibility in Study 1 B.

## Study 1 B

The goal of Study 1 B was to examine the relationship between brand use and Brand Discrimination at a shorter response deadline than that used in the previous study (750 ms vs. 1200 ms). Our primary hypothesis was that, at this shorter response deadline, only users of the Market Leader brand would differ in their Brand Discrimination among the ML brand and its two competitors.

### Method

Participants were 96 members of an online market research panel (29 men, mean age = 40.34, *SD* = 12.19) who participated in return for the chance to win a prize of 300 euros in value. Informed consent was presented in written form at the beginning of the survey. Participants were informed that they would participate in a research study about marketing stimuli, and clicked a radio button indicating their agreement to do so. Only after indicating their agreement were participants allowed to continue to the study. The publicly posted data from this study have been anonymized.

The method was identical to that in Study 1 A, with the one exception that the response deadline for responding to each stimulus was lowered to 750 ms. Internal consistency for the exercises for the Market Leader brand, Competitor 1 and Competitor 2 were .91, .87 and .90, respectively.

### Ethics Statement

In Belgium there is no legal requirement to obtain approval from an institutional review board (IRB) for non-clinical research studies. The authors work in a university in which no IRB existed at the time when the data were collected.

### Results

#### Brand Discrimination Scores

As in Study 1 A, the Brand Discrimination scores were submitted to a repeated-measures ANOVA, with brand (Market Leader vs. Competitor 1 vs. Competitor 2) as a within-subjects factor. Contrary to Study 1 A, this analysis indicated no within-subjects effect of Brand Discrimination, *F* (2, 190) = .42, *p* = .66. Thus, in aggregate, our sample of consumers was unable to discriminate between the brands under study (Table 1). The Brand Discrimination scores were lower in Study 1 B (compared to Study 1 A), which indicates that shortening the response deadline made the task more difficult.

**Table 1:** Means and Standard Deviations for the Brand Discrimination measure for the Market Leader and Competitor brands, Study 1B.

Brand	Brand Discrimination Score (SD)
Market Leader	.86 (.77)
Competitor 1	.85 (.72)
Competitor 2	.80 (.75)

#### Moderation of Brand Discrimination by Brand Use

**Fig 2:**
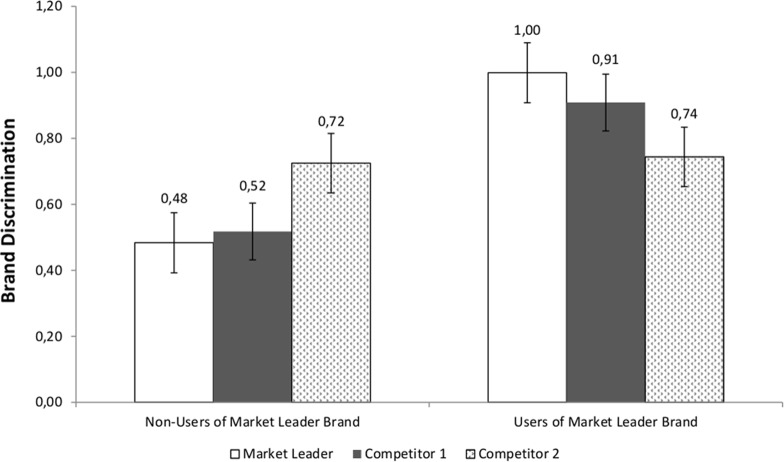
Brand Discrimination for the Market Leader brand and its two competitors for Non-Users and Users of the Market Leader Brand, Study 1 B. Note: Standard error bars are shown for each mean.

We next explored the moderation of the within-subjects Brand Discrimination scores by Market Leader brand use. Specifically, Brand Discrimination scores were submitted to a 3 (Brand Discrimination: Market Leader vs. Competitor 1 vs. Competitor 2) X 2 (Market Leader brand use: yes vs. no) mixed model ANOVA, with repeated-measures on the first factor. As in the previous analysis, the main effect of brand was not statistically significant, *F* (2, 188) = .08, *p* = .93. However, the Brand Discrimination by ML Brand Use interaction was statistically significant, *F* (2, 188) = 5.57, *p* = .004, *ηρ²* = .06, Fig. 2.

This interaction was decomposed by Market Leader brand use. For participants who were not users of the Market Leader brand (*N* = 30), there was no statistically significant within-subjects difference of Brand Discrimination scores for the three brands, *F* (2, 58) = 2.36, *p* = .10.

For users of the Market Leader brand (*N* = 66), however, there was a statistically significant within-subjects difference of Brand Discrimination scores for the three brands, *F* (2, 130) = 3.60, *p* = .03, *ηρ²* = .05. This effect was decomposed, as in Study 1 A, by means of a contrast analysis comparing the Brand Discrimination scores for the Market Leader versus the average Brand Discrimination scores for Competitor 1 and Competitor 2. This contrast was statistically significant, *F* (1, 65) = 4.74, *p* = .03, *ηρ²* = .07. Thus, as hypothesized, at short response deadlines, only users of the Market Leader brand could discriminate among the ML brand and its two competitors. (The complementary orthogonal contrast compared discrimination scores between Competitor 1 and Competitor 2 brands, and it was not statistically significant, *F* (1, 65) = 2.76, *p* = .10.)

### Discussion

The data presented in Study 1 A and Study 1 B provide evidence for the known groups validity of the Brand Discrimination measure by showing that the task can discriminate between a Market Leading brand and two weaker competitor brands. The results of Study 1 B show that, as would be predicted by associative network theories of cognition, at short response deadlines, only users of the Market Leader brand could discriminate among the Market Leader vs. its two competitors. However, it is important to note that the results of Studies 1 A and Studies 1 B are correlational and therefore cannot test the causal relationship between brand exposure and Brand Discrimination

The goal of Study 2 was to build upon the results of Studies 1 A and 1 B and to provide further evidence for the validity of the Brand Discrimination task. Therefore, in Study 2 we used an experimental methodology to manipulate the strength of mental representations of a given brand and measured Brand Discrimination for that brand and another in the same category. We then examined whether our experimental manipulation of brand exposure influenced Brand Discrimination scores for the exposed vs. non-exposed brands.

## Study 2

The goal of Study 2 was to provide experimental evidence for the validity of the Brand Discrimination measure. This study is guided by the discussion of the concept of validity developed by Borsboom and colleagues [39], who advocate using experimental methodology to demonstrate validity of measurement instruments. According to these authors, “a test is valid for measuring an attribute if variation in the attribute causes variation in the test scores” (pp. 1067). This experimental approach to validity has also been recommended explicitly for the validation of implicit measures [29,38]. We contend that the Brand Discrimination task measures the strength of mental associations of a brand with its defining elements. Therefore, in Study 2, we manipulated the strength of mental associations of a given brand and its elements, and tested the effect of this manipulation on subsequent scores of the Brand Discrimination measure.

There were three experimental conditions in this study. In all conditions, participants were first exposed to a series of images. In the Control condition, participants were exposed to neutral, non-brand related images. In the Simple Exposure condition, participants were exposed to images depicting the brand elements (e.g. logo, color) of two bottled water brands (Evian and San Pellegrino), and were instructed simply to observe the images. In the Active Reinforcement Condition, participants were exposed to images depicting the brand elements of the same two water brands (Evian and San Pellegrino), and were required to actively identify the brand for each image that was presented on the screen. Following this first task, participants completed a Brand Discrimination exercise for two brands: Evian and Vittel. Our primary hypothesis was that brand discrimination for the Evian brand would be higher in the Simple Exposure and Active Reinforcement conditions as compared to the control condition. We expected no significant variation in the Brand Discrimination scores for Vittel across experimental conditions.

### Pre-test

In Study 2, we examined brands in the bottled water category. The brands used in this study were Evian, Vittel (a French water brand), Spa (a Belgian water brand), and San Pellegrino (an Italian water brand). For each of these brands, we chose four different visual marketing elements for each brand: logos, colors, bottle shape, and brand visuals (an advertising image evocative of the brand architecture; see S1 Fig. and S2 Fig. for the brand stimuli). In order to ensure that the stimuli chosen were perceived as relating to their parent brand, we conducted a pre-test.

Participants (*N* = 25) evaluated all brand images on 7-point scales (from *not at all* to *very much*), rating the extent to which each image was related to each of the three brands under study. Results revealed that participants rated the images as belonging more to the appropriate brand than for the other three, F’s for Evian, Vittel, Spa and San Pellegrino, of *F* (3, 72) = 114.68, *p* < .001, *F* (3, 72) = 62.86, *p* < .001, *F* (3, 72) = 467.88, *p* < .001, *F* (3, 72) = 186,18, *p* < .001, respectively.

### Method

Participants were 164 students who participated in return for partial course credit (85 men, mean age = 20.56, *SD* = 1.61). Informed consent was oral. Participants were told that they would participate in a research study about marketing stimuli, and that they were free to withdraw at any time without penalty. All participants agreed to participate; none withdrew during the study. Only after giving oral consent was each participant allowed to begin the study. Following the study, all participants were thoroughly debriefed. They were further given the opportunity to ask any questions they had about the research. The publicly posted data from this study have been anonymized.

The study design conformed to a 3 (condition: Control vs. Simple Exposure vs. Active Reinforcement) by 2 (Brand Discrimination: Evian vs. Vittel) design, with condition as a between-subjects factor and Brand Discrimination as a within-subjects factor. The study was conducted in a university laboratory setting. The Brand Discrimination exercise was presented on 40 cm VGA computer monitors operating at a resolution of 1024 X 768 pixels. Computers were running Windows 7 and the default color settings for the monitors and for the operating system were used. The room was lit with ambient light from overhead fluorescent light bulbs. The observation distance was approximately 55 cm and the visual angle subtended (calculated via the formula presented by [40]) was approximately 23 degrees. The experiment was run using the Affect 4.0 software package [41].

In all conditions, participants first were exposed to a series of 96 images; each image was presented for 2500 ms in the center of a computer screen. The inter-stimulus interval between each trial was 1000 ms. The images were presented in 2 blocks of 48 trials. Participants were instructed to observe the images that appeared on the screen. The selection of images presented (and the instructions) constituted the experimental manipulation. In the Control condition, participants were exposed to a series of non-brand-related stimuli in the form of pictures of buildings.

In the Simple Exposure condition, participants were exposed to a series of brand images. The brand images consisted of the four brand elements for Evian and San Pellegrino, as described above. Each brand image was presented three times each in a random order. Participants were instructed simply to observe the images as they were presented on the screen. This manipulation was designed to strengthen the links between the parent brand and its elements without requiring an active response on the part of the participants (see [42] for a conceptually similar approach).

In the Active Reinforcement condition, participants were exposed to the same series of brand images as in the Simple Exposure condition. However, in this condition, participants were additionally instructed to identify the brand of the images displayed on the screen. Participants identified the brand for each image by pressing either the A or the P key on an AZERTY keyboard (the brand to which each key corresponded- either Evian or San Pellegrino- was counter-balanced across participants). This manipulation was designed to actively strengthen the mental links between the parent brand and its elements (see [43,44] for similar approaches).

Following the experimental manipulation, all participants completed a Brand Discrimination exercise which consisted of a single block of 48 trials. The target brands in the task were Evian and Vittel; Spa served as a distracter brand. The target present and target absent trials for Evian and Vittel were presented in a different random order for each participant. Each of the four brand elements for Evian and Vittel was presented three times in conjunction with the target brand, resulting in a total of 12 target present trials for each brand. Twelve target absent trials were included for each brand, pairing each target brand name with a randomly selected image from the other two brands. The response deadline for all trials was 1000 ms. Internal consistency for the Brand Discrimination exercises for Evian and Vittel were .69 and .72, respectively.

### Ethics Statement

In Belgium there is no legal requirement to obtain approval from an institutional review board (IRB) for non-clinical research studies. The authors work in a university in which no IRB existed at the time when the data were collected.

### Results

**Fig 3:**
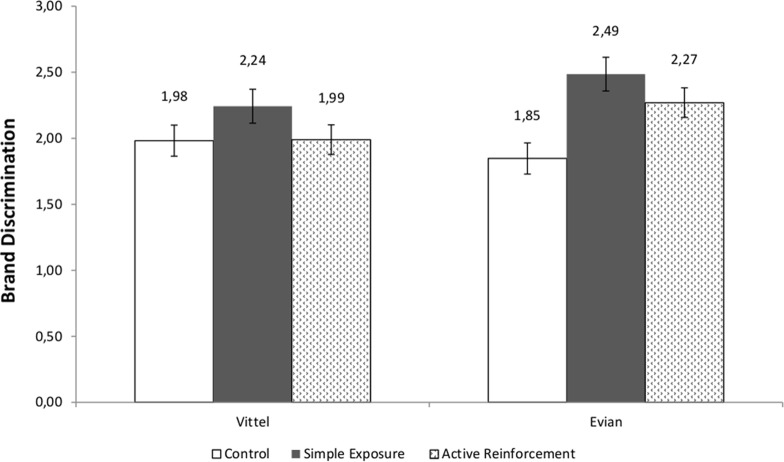
Brand Discrimination scores for Vittel and Evian as function of the Experimental Condition, Study 2. Note: Standard error bars are shown for each mean.

We examined the Brand Discrimination scores for Evian and Vittel in a 3 (condition: Control vs. Simple Exposure vs. Active Reinforcement) by 2 (Brand Discrimination: Evian vs. Vittel) mixed model ANOVA, with repeated-measures on the first factor. This analysis indicated a marginal within-subjects effect of Brand Discrimination *F* (1, 161) = 3.77, *p* = .054, *ηρ²* = .02, which was qualified by a significant condition by Brand Discrimination interaction *F* (2, 161) = 3.90, *p* = .02, *ηρ²* = .05, see Fig. 3.

This interaction was decomposed by brand. The Brand Discrimination scores for each brand were submitted to a one-way ANOVA, with condition (Control vs. Simple Exposure vs. Active Reinforcement) as a between-subjects independent variable.

For the Evian Brand Discrimination scores, the brand that had been viewed by participants in Part 1 of the study, there was a statistically significant effect of condition, *F* (2, 161) = 7.66, *p* = .001, *ηρ²* = .09. Follow up contrast analyses revealed that, as hypothesized, Evian Brand Discrimination scores were significantly higher the Simple Exposure condition (*M* = 2.49, *SD* = .71) and in the Active Reinforcement condition (*M* = 2.27, *SD* = .87), as compared to the control condition (*M* = 1.85, *SD* = .96).

For the Vittel Brand Discrimination scores, there was no effect of experimental condition *F* (2, 161) = 1.37, *p* = .26, indicating no statistically significant variation among the Brand Discrimination scores in the Control (*M* = 1.98, *SD* = .91), Simple Exposure (*M* = 2.24, *SD* = .88) and Active Reinforcement conditions (*M* = 1.99, *SD* = .99).

### Discussion

This study was designed to provide experimental evidence for the validity of the Brand Discrimination measure. We expected that participants who were directly exposed (either passively or actively) to Evian brand images in Part 1 of the study would have stronger mental representations of that brand, and therefore evidence higher Brand Discrimination scores for Evian (but not Vittel) in Part 2 of the study. This hypothesis was supported. Participants in the Simple Exposure and Active Reinforcement conditions evidenced higher Brand Discrimination scores for Evian, compared to participants in the Control condition. The Brand Discrimination scores for Vittel, in contrast, did not significantly vary as a function of experimental condition. The Brand Discrimination task therefore meets the definition of validity discussed by Borsboom and colleagues [36], and explicitly recommended for demonstrating the validity of implicit tasks [29,38]: after manipulating the strength of the mental associations of a specific brand and its elements, Brand Discrimination scores for the manipulated brand increased, compared to a control group who received no such manipulation.

## Study 3

The goal of Study 3 was to provide evidence of stability across time for the Brand Discrimination task. To this end, we conducted an investigation of test-retest reliability. Participants completed the Brand Discrimination task for two different brands at two different time points, and the stability of the Brand Discrimination scores across time was assessed.

### Method

Participants were 133 students who participated in return for partial course credit (52 men, mean age = 20.33, *SD* = 1.95). Two research sessions, separated by 3 weeks, were conducted in the same university laboratory setting as Study 2. Participants created an anonymous ID number (the last 6 digits of their phone number) and used this ID number at both testing sessions. The ID number was subsequently used to match the scores from each participant at Session 1 and Session 2. Informed consent was oral. At each session, participants were told that they would participate in a research study about marketing stimuli, and that they were free to withdraw at any time without penalty. All participants agreed to participate; none withdrew during the sessions. Only after giving oral consent was each participant allowed to begin each session. Following Session 2, all participants were thoroughly debriefed. They were further given the opportunity to ask any questions they had about the research. The publicly posted data from this study have been anonymized.

At both sessions, participants completed the same Brand Discrimination task used in Part 2 of Study 2. This exercise uses Evian and Vittel as target brands, while Spa serves as a distracter brand. Internal consistencies for Evian and Vittel at Session 1 were .71 and .79, respectively. Internal consistencies for Evian and Vittel at Session 2 were .80 and .81, respectively. Examination of histograms, skewness and kurtosis, and qq-plots suggested that the Brand Discrimination scores were approximately normally distributed.

### Ethics Statement

In Belgium there is no legal requirement to obtain approval from an institutional review board (IRB) for non-clinical research studies. The authors work in a university in which no IRB existed at the time when the data were collected.

### Results

The test-retest correlations of the Brand Discrimination scores, along with their means and standard deviations, are shown in Table 2. The test-retest correlation for the Evian Brand Discrimination scores was .58, and the test-retest correlation for the Vittel Brand Discrimination scores was .64, both statistically significant at *p* < .001. Bland-Altman Plots for the test-retest measurements of Evian and Vittel Brand Discrimination are presented in S5 Fig. and S6 Fig.

**Table 2:** Test-retest Correlations, Means and Standard Deviations for the Brand Discrimination Scores for the two testing sessions, Study 3.

		Time 1		Time 2	
Brand Discrimination	*Test-Retest Correlation*	*M*	*SD*	*M*	*SD*
Evian	.58 **	1.49	.95	2.06	1.06
Vittel	.64 **	1.29	1.06	2.02	1.11

*Note*: ** p <. *001*.

### Discussion

This study provides evidence for stability across time of the Brand Discrimination measure. By way of comparison, a 2007 review [45] (see also [46]) examined work done up until that time and found that test-retest correlations in the literature for the Implicit Association test (e.g. the IAT) ranged between .32 to .69, with a median value of .56; test-retest correlations were independent of the time separating the first and second testing occasions. A recent paper on the IAT and drinking [47] examined the test-retest reliability (with a one week time window) of 6 different IAT’s related to alcohol among 39 individuals. The test-retest correlations ranged from .27 to .70, with a median value of .40. From this perspective, the test-retest correlations for the Brand Discrimination Index compare favorably with those from the IAT.

It should be noted that the Brand Discrimination scores increased for both brands at the second measurement occasion. This within-participant shift in mean values across repeated testing occasions has also been documented for implicit attitude measures such as the IAT [32,48]. In the current study, the increase in Brand Discrimination scores at Time 2 could be due to increased familiarity with the structure of the task, increased familiarity with the brand stimuli, or both, at the second (compared to the first) testing occasion (see [49]). It should be noted that such learning effects might also be expected with test-retest examinations of explicit measures of brand association, for example questionnaire measures which require participants to identify or match brand stimuli with their parent brand.

## Study 4

The goal of Study 4 was to demonstrate the predictive validity of the Brand Discrimination measure. Indeed, based on previous theory and research, the strength of mental representations of brands should be predictive of brand choice [6,19]. Study 4 was designed to test this hypothesis. We measured Brand Discrimination and two explicit questionnaire measures of brand evaluation, and tested whether these constructs predicted brand choice, both independently and when entered simultaneously as predictors in a logistic regression analysis.

### Pre-test

In Study 4, we examined brands in the sportswear category. The target brands used in this study were Adidas and Nike, with Puma serving as a distracter brand in the Brand Discrimination exercises. For each of these brands, we chose 5 visual marketing elements: logos, branded jogging shorts, t-shirts, baseball hats, and shoeboxes (see S3 Fig. for the brand stimuli).

In order to confirm that the stimuli we chose are in fact perceived as representative of the given brand, we conducted a pre-test. Participants (*N* = 26) evaluated all brand stimuli on 7-point scales (from *not at all* to *very much*), rating the extent to which each stimulus was related to each of the three brands under study. Results revealed that participants rated the images as belonging more to the appropriate brand than for the other two, F’s for Adidas, Nike and Puma of *F* (2, 50) = 1214.94, *p* < .001, *F* (2, 50) = 1661.37, *p* < .001, F (2, 50) = 1075.57, *p* < .001, respectively.

### Method

Participants were 97 members of an online market research panel (50 men, mean age = 42.18, *SD* = 11.91) who participated in return for the chance to win a prize. Informed consent was presented in written form at the beginning of the survey. Participants were informed that they would participate in a research study about marketing stimuli, and clicked a radio button indicating their agreement to do so. Only after indicating their agreement were participants allowed to continue to the study. The publicly posted data from this study have been anonymized.

Participants first completed Brand Discrimination exercises for the Adidas and Nike brands. The task was presented in two blocks of trials, with a single brand (either Adidas or Nike) serving as a target brand in each block. Each block contained a total of 15 target present trials (the 5 target brand elements shown 3 times each) and 15 target absent trials (with brand elements randomly chosen from the non-target brand and Puma). Block order was counter-balanced across participants. The response deadline for responding to each stimulus was 1000 ms. Internal consistencies for the Brand Discrimination exercises for Adidas and Nike were .94 and .95, respectively.

Following the Brand Discrimination exercises, participants responded to a number of explicit questionnaire measures about the focal brands (Adidas and Nike), with brand order counter-balanced across participants. For each brand, participants completed a 3-item index of brand attitude (e.g. “Adidas is a favorable brand”, “Adidas is a likeable brand” and “Adidas is a pleasing brand” [50]) and a 3-item index of brand affect (e.g. “I feel good when I use Adidas products”, “The brand Adidas makes me happy”, and “The brand Adidas gives me pleasure”[51]), with all items answered on 7 point Likert scales. Internal consistencies for the brand attitude and affect scales for Adidas were .88 and .84, respectively. Internal consistencies for the brand attitude and affect scales for Nike were .86 and .89, respectively.

Finally, participants were reminded that their participation entered them into a lottery to win a prize. Participants were asked from which brand, either Adidas or Nike, they would prefer to receive a prize, if they were to win the lottery. This served as our index of brand choice. After the study was completed, a prize winner was chosen at random from among all participants and was given a prize from their chosen brand.

### Ethics Statement

In Belgium there is no legal requirement to obtain approval from an institutional review board (IRB) for non-clinical research studies. The authors work in a university in which no IRB existed at the time when the data were collected.

### Results

We first created relative indexes for the Brand Discrimination and explicit measures by subtracting the Nike scores for each construct from the Adidas scores for that construct (e.g. Relative Brand Discrimination for each participant was computed as: Adidas Brand Discrimination score—Nike Brand Discrimination score for that participant). For analysis, Brand Choice was coded as 0 = Nike, 1 = Adidas.

The correlation matrix of these variables is presented in Table 3.

**Table 3:** Correlations among Brand Choice and the Relative Brand Variables, Study 4.

	Choose Adidas	Relative Brand Discrimination	Relative Brand Attitude	Relative Brand Affect
Choose Adidas	-	.23 *	.48 **	.45 **
Relative Brand Discrimination		-	.06	.03
Relative Brand Attitude			-	.66 **
Relative Brand Affect				-

*Note*:

* p <. *05*,

** p <. *001*.

Consistent with our hypothesis, the Brand Discrimination was related to brand choice, as were both of brand attitude and brand affect. The explicit measures evidenced higher correlations with choice than did Brand Discrimination. Brand attitude and brand affect were highly correlated with one another, but neither was correlated with Brand Discrimination.

We then conducted a logistic regression analysis, predicting brand choice as a function of the relative Brand Discrimination, brand attitude and brand affect variables (Table 4). All predictors explained unique variation in brand choice. The Nagelkerke R Square for the model was 44%.

**Table 4:** Logistic Regression Analysis Predicting Brand Choice, Study 4.

	B	S.E.	Wald	df	p	Exp(B)
Intercept	.38	.28	1.83	1	.18	1.47
Relative Brand Discrimination	.70	.31	5.14	1	.02	2.01
Relative Brand Attitude	.28	.12	5.61	1	.02	1.32
Relative Brand Affect	.22	.11	4.20	1	.04	1.24

### Discussion

Study 4 examined the relationships between Brand Discrimination, explicit brand attitude and affect, and brand choice. The results revealed that Brand Discrimination and the explicit measures were all correlated with brand choice. Furthermore, when examining the measures together in a logistic regression, all predicted unique variance in brand choice. This suggests that the Brand Discrimination measure provides information that is useful to predict brand choice above and beyond what is captured by reflective explicit questionnaire measures of brand evaluation. This finding is consistent with the notion that the strength of consumers’ mental representations of brands can be a key component of consumer-based brand equity [6,13].

## Study 5

The goal of Study 5 was to provide further evidence of the validity of the Brand Discrimination index in 3 primary ways. The first was to offer a replication of the relationships found in Study 4, by showing that Brand Discrimination can predict brand choice with different brands in a different category (consumer durables in the form of cell phones). The second was to determine the relationships with implicit brand attitudes, which measure both accessibility and valence (positive/negative) of brand associations. The third was to investigate the discriminant validity of the Brand Discrimination measure in predicting brand choice when controlling for implicit attitude measures.

Participants in this study completed the Brand Discrimination task for two cell phone brands, along with the Go/No-Go Association Task (e.g. the GNAT; [34]), which measures positive and negative implicit attitudes, for each brand. Participants also completed explicit questionnaire measures of attitude and affect for each brand. Finally, as a measure of brand choice, participants were asked to allocate virtual lottery tickets to receive a prize from one of the brands under study. Our primary hypothesis was that Brand Discrimination scores would, as in Study 4, predict the allocation of lottery tickets (an index of brand choice).

### Pre-tests

In Study 5, we examined brands in the cell phone category. The target brands used in this study were Nokia and Sony Ericsson, with the Apple Iphone serving as a distracter brand in the implicit exercises. For each of these brands, we identified 4 visual marketing elements: logos, colors, an “iconic” phone model (e.g. with design characteristics that were typical for the brand), and the brand slogan (e.g. “Connecting People” for Nokia and “Make Believe” for Sony Ericsson, see S4 Fig. for the brand stimuli).

In order to confirm that the images selected are indeed seen as representative of the given brand, we conducted a pre-test. Participants (*N* = 34) evaluated all images on 7-point scales (from *not at all* to *very much*), rating the extent to which each image was related to each of the three brands under study. Results revealed that participants rated the images as belonging more to the appropriate brand than for the other two, F’s for Nokia, Sony Ericsson and IPhone of *F* (2, 66) = 339.98, *p* < .001, *F* (2, 66) = 439.54, *p* < .001, *F* (2, 66) = 351.76, *p* < .001, respectively.

The Go/No-Go Task presents stimuli to participants, and requires them to judge each stimulus in regards to a target category (brand in the current study) and an attribute dimension (e.g. positive or negative when measuring implicit attitudes). In the current study, we used brand visuals to represent the target (e.g. brand) categories and emoticons to represent positive and negative attributes. We chose 3 different positive emoticons to represent positive attributes, and 3 different negative emoticons to represent negative attributes.

In order to confirm that the chosen emoticons represented the desired attribute valence, we conducted a pre-test. Participants (*N* = 19) evaluated each of the 6 emoticons on two 7-point scales (from *not at all* to *very much*), rating the extent to which each emoticon was positive or negative, respectively. Results indicated that participants rated the positive emoticons as more positive than negative, *F* (1, 18) = 479.16, *p* < .001. Conversely, negative emoticons were rated as more negative than positive, *F* (1, 18) = 286.38, *p* < .001.

### Method

Participants were 112 members of an online market research panel (62 men, mean age = 49.95, *SD* = 14.36) who participated in return for the chance to win a prize. Informed consent was presented in written form at the beginning of the survey. Participants were informed that they would participate in a research study about marketing stimuli, and clicked a radio button indicating their agreement to do so. Only after indicating their agreement were participants allowed to continue to the study. The publicly posted data from this study have been anonymized.

Participants first completed two blocks of implicit tasks: one block contained the Brand Discrimination exercise for both Nokia and Sony Ericsson, and the other block contained the Go / No-Go Association Task exercises to measure positive and negative implicit attitudes towards both Nokia and Sony Ericsson. The order of the implicit tasks (Brand Discrimination vs. GNAT) was counter-balanced across participants.

The Brand Discrimination exercise consisted of a single block of 48 trials. The target brands in the task were Nokia and Sony Ericsson; the IPhone served as a distracter brand. The target present and target absent trials for Nokia and Sony Ericsson were presented in a different random order for each participant. Each of the four brand elements for Nokia and Sony Ericsson was presented three times in conjunction with the target brand, resulting in a total of 12 target present trials for each brand. Twelve target absent trials were included for each brand, pairing each target brand name with a randomly selected image from the other two brands. The response deadline for all trials was 1000 ms. Internal consistencies for the Brand Discrimination indices for Nokia and Sony Ericsson were .70 and .75.

The GNAT was administered in a single block of 168 trials. Each implicit evaluative attribute (e.g. positive and negative implicit attitude) for each brand was assessed via 42 trials, in which participants classified either a brand image or a positive/negative emoticon as belonging to a brand or an evaluative category. For example, to measure positive implicit attitude for Nokia, a total of 42 trials contained the header Nokia + Positive. Half of these were target present trials: 12 were Nokia stimuli (each of the 4 brand elements repeated 3 times) and 9 were positive emoticons (each of the 3 positive emoticons presented 3 times each). The remaining half were target absent trials: 12 were brand stimuli randomly selected from the other two brands, and 9 were negative emoticons (each of the 3 negative emoticons presented 3 times each). The response deadline was 1000 ms for all trials, and trial order was randomly determined for each participant. Pictorial versions of the GNAT have been used in previous research [34,52]. The GNAT was scored using the algorithm described by Nosek and Banaji [34]. Internal consistencies for the positive and negative GNAT for Nokia were .91 and .90, respectively. Internal consistencies for the positive and negative GNAT for Sony Ericsson were .92 and .89, respectively.

Following the implicit tasks, participants completed questionnaire measures of brand attitude and brand affect for Nokia and Sony Ericsson, using the same scales employed in Study 4. Brand and dimension (attitude and affect) were counter-balanced across participants. Internal consistencies for the brand attitude and affect scales for Nokia were .96 and .94, respectively. Internal consistencies for the brand attitude and affect scales for Sony Ericsson were .95 and .94, respectively.

After responding to the brand questionnaires, participants completed the brand choice lottery ticket allocation. All participants were reminded that in return for their participation, they would be entered into a lottery to win a prize. Participants were given 5 virtual “lottery tickets”, which they could use to increase their chances of getting the prize they wanted. Participants could allocate their lottery tickets to one of two prizes: a Nokia phone worth 300 euros, or a Sony Ericsson phone worth 300 euros. To prevent strategic ticket allocation, participants were additionally told: “prize winners will be chosen for each mobile phone when that lottery accumulates 1000 lottery tickets. Therefore, each ticket gives you an equal chance to win the prize of your choosing.”

We calculated the percentage of tickets each participant allocated to the brand Nokia. This results in an index with 6 options (0%, 20%, 40%, 60%, 80% or 100%), which we recoded as a 0-to-5 scale for the following analyses. Higher scores indicate a greater relative preference for Nokia vs. Sony Ericsson, respectively.

### Ethics Statement

In Belgium there is no legal requirement to obtain approval from an institutional review board (IRB) for non-clinical research studies. The authors work in a university in which no IRB existed at the time when the data were collected.

### Results

We first created relative scores for the Brand Discrimination and explicit measures by subtracting the Sony Ericsson values for each construct from the Nokia values for that construct (e.g. Relative Brand Discrimination for each participant was computed as: Nokia Brand Discrimination score—Sony Ericsson Brand Discrimination score for that participant).

The correlation matrix of these variables, along with lottery ticket allocation, is presented in Table 5.

**Table 5:** Correlations among Lottery Ticket Allocation (Brand Choice) and the Relative Brand Variables, Study 5.

	Nokia Tickets	Relative Brand Discrimination	Relative Positive Implicit Attitude	Relative Negative Implicit Attitude	Relative Explicit Attitude	Relative Affect
Nokia Tickets	-	.26 **	.09	-.045	.50 **	.53 **
Relative Brand Discrimination		-	.15	.10	.18	.14
Relative Positive Implicit Attitude			-	.34 **	.18	.20 *
Relative Negative Implicit Attitude				-	.05	.07
Relative Explicit Attitude					-	.89 **
Relative Affect						-

*Note*:

* p <. *05*,

** p <. *01*.

As in Study 4, the Brand Discrimination measure, along with attitude and affect, all evidenced statistically significant relationships with brand choice. Brand attitude and affect were again stronger predictors of brand choice than was the Brand Discrimination measure. Neither the positive nor the negative GNAT score was related to brand choice.

All the above measures were used in a linear regression to predict the number of tickets attributed to the brand Nokia. Due to the high correlation between attitude and affect (*r* = .89), we summed these variables to create a single relative explicit evaluation variable (including both attitude and affect in the regression analysis leads to multicollinearity and VIF’s > 4 for both variables).

The results of this analysis are shown in Table 6. Both the Brand Discrimination measure and the explicit brand measure significantly predicted brand choice. The R square for the regression model was 32%.

**Table 6:** Linear Regression Analysis Predicting Lottery Ticket Allocation (Brand Choice), Study 5.

	b	Std. Error	*β*	t	p
Intercept	2.76	.12		23.31	.00
Relative Brand Discrimination	.32	.15	.18	2.24	.03
Relative Positive Implicit Attitude	.00	.18	.00	.02	.99
Relative Negative Implicit Attitude	-.22	.20	-.10	-1.12	.27
Relative Explicit Measurement (Attitude and Affect)	.09	.02	.50	6.12	.00

### Discussion

The findings from Study 5 provide a replication and extension of those from Study 4. As in Study 4, both the Brand Discrimination measure and the explicit questionnaire measure predicted brand choice. Implicit attitude, as measured by the GNAT, was unrelated to brand choice. The results of Study 5 provide further evidence for the discriminant validity of the Brand Discrimination task by demonstrating that it provides information relevant to predicting brand choice above and beyond what is captured by implicit positive and implicit negative attitudes.

## General Discussion

This article describes and validates an implicit measure, which we call the Brand Discrimination task, to understand consumers’ mental representations of a brand’s marketing elements. Studies 1 A and 1 B showed that the task can discriminate among strong and weak brands, and that at shorter response deadlines, only brand users were able to discriminate among a Market Leading brand and two weaker competitors. Study 2 provided experimental evidence for the validity of the Brand Discrimination measure. Specifically, we manipulated the strength of participants’ mental representation of brand elements (the underlying attribute the task measures), and showed that Brand Discrimination scores varied as a function of the manipulation. Study 3 showed that the Brand Discrimination measure is relatively stable across time, comparing favorably with published figures for test-retest reliability of the IAT, another widely-used implicit measure. Studies 4 and 5 demonstrate the predictive validity of the Brand Discrimination task. In both studies, Brand Discrimination scores predicted brand choice, both independently and controlling for other explicit and implicit measures.

### Theoretical and Methodological Contributions

The current research provides empirical support for a number of previous theoretical contentions about consumer representations of brands in memory. Numerous scholars have described brands as networks of brand associations, and argued for the importance of understanding the mental representations of brand knowledge in consumer memory [6,13–15,25,53]. Our research is consistent with the notion that strong and unique brand associations are a key part of customer-based brand equity [6,13,17,24], in that Brand Discrimination scores are higher for strong (vs. weak) brands, are relatively stable across time, and are predictive of brand choice.

However, the current research also goes beyond existing work in several key ways. While a number of qualitative and questionnaire-based techniques have been developed to understand consumer’s mental brand associations [5,15,25,53], these types of assessment are at best approximations of the strength of brand representations in memory [53]. In contrast, the Brand Discrimination task described in the current paper was guided by social cognitive research techniques to more directly tap the strength of consumers’ mental representations of a brand’s marketing elements. Compared to previous efforts, our Brand Discrimination measure offers advantages in the standardization of application, measurement and scoring of the task, and provides a quantitative index which we demonstrate corresponds to brand strength and mental representations of brands and their elements.

### Managerial Contributions

The current research also has implications for the practice of marketing and brand management. First, the current work provides empirical support to the notion that the strength and uniqueness of brand associations are a key part of consumer-based brand equity, and that understanding mental relationships for one’s brand, in comparison to competitors, is important [6]. The current research can contribute to marketing practice by providing a tool which permits marketers and brand managers to do precisely that. By studying consumers’ Brand Discrimination scores for their own and competitors’ brands, companies can gain insight into the strength of the brand associations for their own versus competitors’ brands. Furthermore, Brand Discrimination exercises can be included in tracking studies (e.g. regularly-conducted market research investigations [6]) to follow the evolution of brand strength across time. Employed in this manner, the task can also be used to assess the success of marketing campaigns or actions; one criterion upon which the success of a marketing campaign can be assessed is whether consumers’ Brand Discrimination scores vary as a function of the brand’s marketing actions.

Second, the current findings provide knowledge which can help brand managers and marketers engage in efficient communication strategies to help cement their brands in the minds of consumers. A key presumption of much advertising and marketing communication is that exposure to brand stimuli can help strengthen consumers’ mental representation of brands [6,13]. In Study 2, we provide evidence that both passive and active exposure to brand stimuli helps strengthen their links with the parent brand in the minds of consumers. This finding suggests that marketers should consistently include key brand elements (e.g. colors, logos, imagery) in marketing communications; doing so should increase the strength and uniqueness of the consumers’ mental associations of marketing elements with the brand.

### Limitations and Caveats

This work is not without limitations. A first limitation of the current studies is that we did not measure participants’ visual acuity. It is clear that individuals who cannot see or who have very poor eyesight would be unable to perform well in a Brand Discrimination exercise, as would also be the case for any other methodology which relies on measuring responses to visual stimuli presented for relatively short durations on a computer screen. It is perhaps worth noting that this situation mimics the real-life situation that marketers and brand managers face: they must create visual brand elements for all members of the public, including those with reduced visual acuity. While the results of the current research suggest that the Brand Discrimination task is capable of detecting differences in brand recognition, and therefore appears robust against this potential limitation, future work could fruitfully examine the moderation of Brand Discrimination by visual acuity.

Second, Studies 1, 4 and 5 were conducted online, with the result that there was variation in the technical specifications (such as color settings) of the computer monitors participants used to complete the Brand Discrimination tasks. While this variation is a potential source of variation in the data, the results of these studies are consistent with those of Studies 2 and 3 (conducted in a laboratory setting with identical computer monitors for all participants), suggesting that the findings presented in the current paper are robust against this potential limitation. It is worth noting that researchers have found robust effects for the Stroop task (a reaction-time identification task for which accurate color representation is critical) when administering the task online (e.g. in the same circumstances in which we conducted our research, [54]), and even when using a “Stroop app” for smartphones and tablets [55]. Thus, the both our findings and those of other researchers suggest that potential variation in technical specifications (for example, with color presentation) across participants’ computer monitors does not alter the results one obtains when administering reaction-time based identification tasks online.

Third, the Brand Discrimination score is a signal-detection-theory-derived index of recognition accuracy of a brand’s marketing elements in the context of those of its competitors. Therefore, changing the marketing elements used to study a given brand will likely change its overall recognition rate, and thus its Brand Discrimination Score. The same would be true of any recognition test; when changing a brand’s to-be-recognized elements, one likely changes the overall recognition score for that brand. The advantage of this approach is its flexibility in accommodating the diverse nature and number of each brand’s visual marketing elements. The disadvantage of this approach is that, if one studies the same brand in two different studies but uses different brand stimuli to represent the brand in each study, the resulting Brand Discrimination Scores do not reflect the exact same quality. Therefore, when conducting multiple studies of a given brand with the Brand Discrimination task, it is important to ensure that the tested stimuli for that brand are the same in each study.

## Conclusion

In conclusion, many scholars have argued for the importance of consumer mental representations of brands as a key component of consumer-based brand equity. The current research provides unique data to support this contention, and describes and validates a measurement tool to quantify this important consumer-based brand attribute.

## Supporting Information

S1 Fig Brand Stimuli, Study 2. (TIF)

S2 Fig Brand Stimuli, Study 2. (TIF)

S3 Fig Brand Stimuli, Study 4. (TIF)

S4 Fig Brand Stimuli, Study 5. (TIF)

S5 Fig Bland-Altman Plot: Evian Brand Discrimination. (PNG)

S6 Fig Bland-Altman Plot: Vittel Brand Discrimination. (PNG)
