# Supplementary figures and images for: Brand Discrimination: An Implicit Measure of the Strength of Mental Brand Representations
Source: PLoS One. 2015 Mar 24;10(3):e0121373. doi: 10.1371/journal.pone.0121373 (PMC4372428; doi:10.1371/journal.pone.0121373)

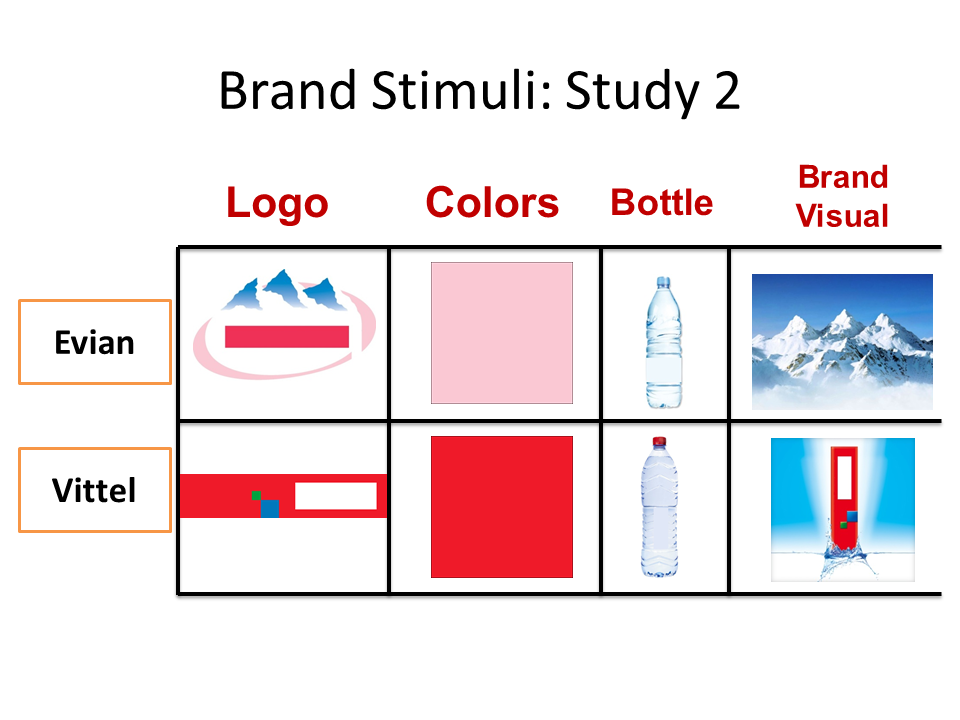

Supplement: S1 Fig — (TIF) [file pone.0121373.s001.tif]

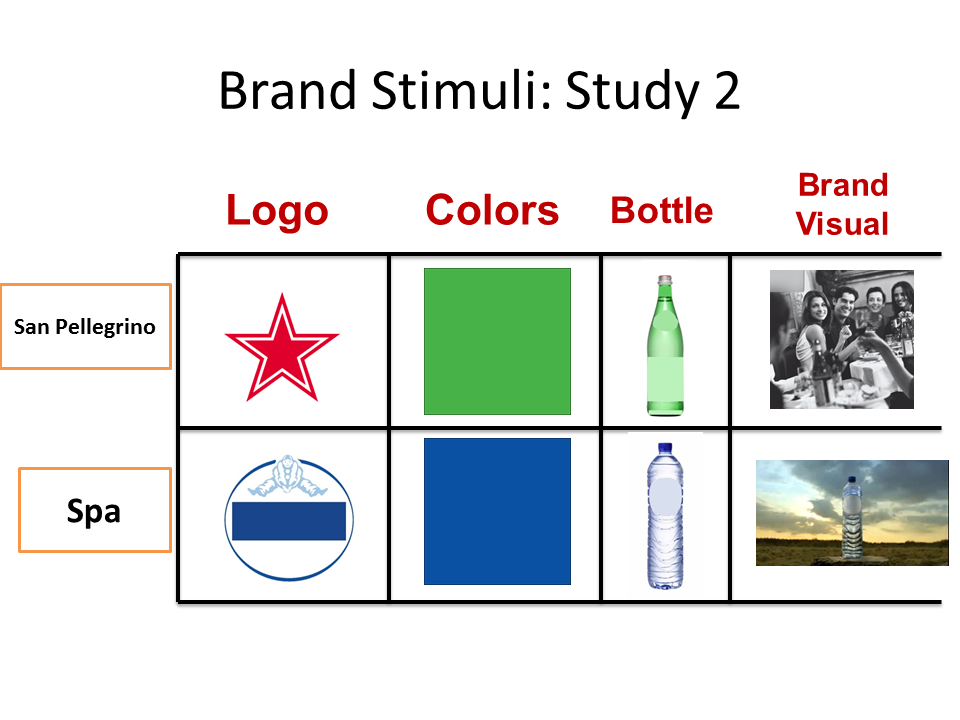

Supplement: S2 Fig — (TIF) [file pone.0121373.s002.tif]

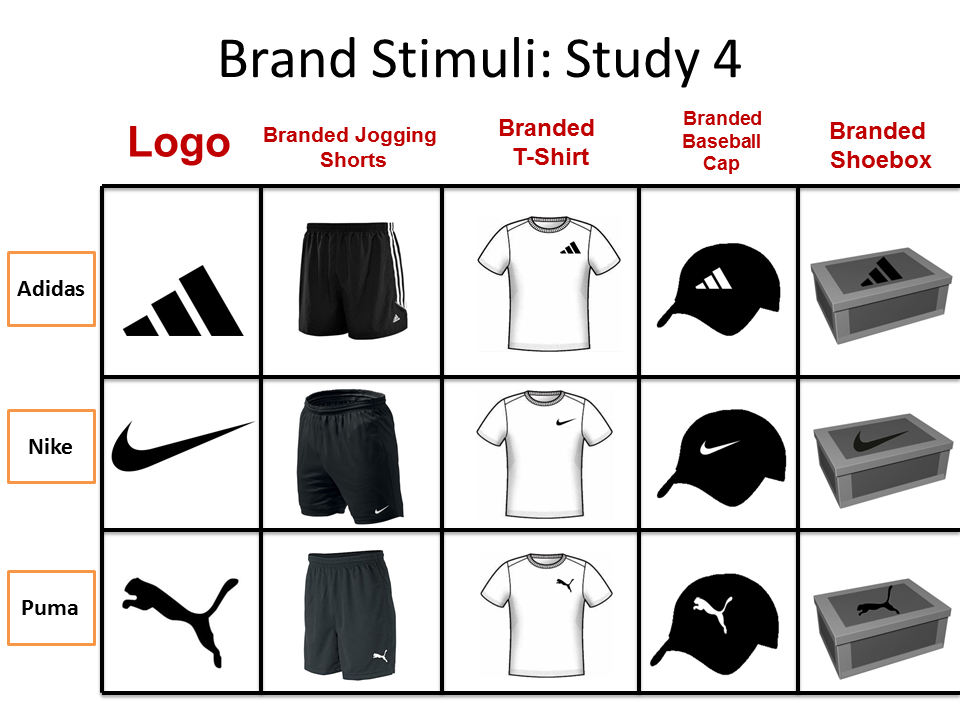

Supplement: S3 Fig — (TIF) [file pone.0121373.s003.tif]

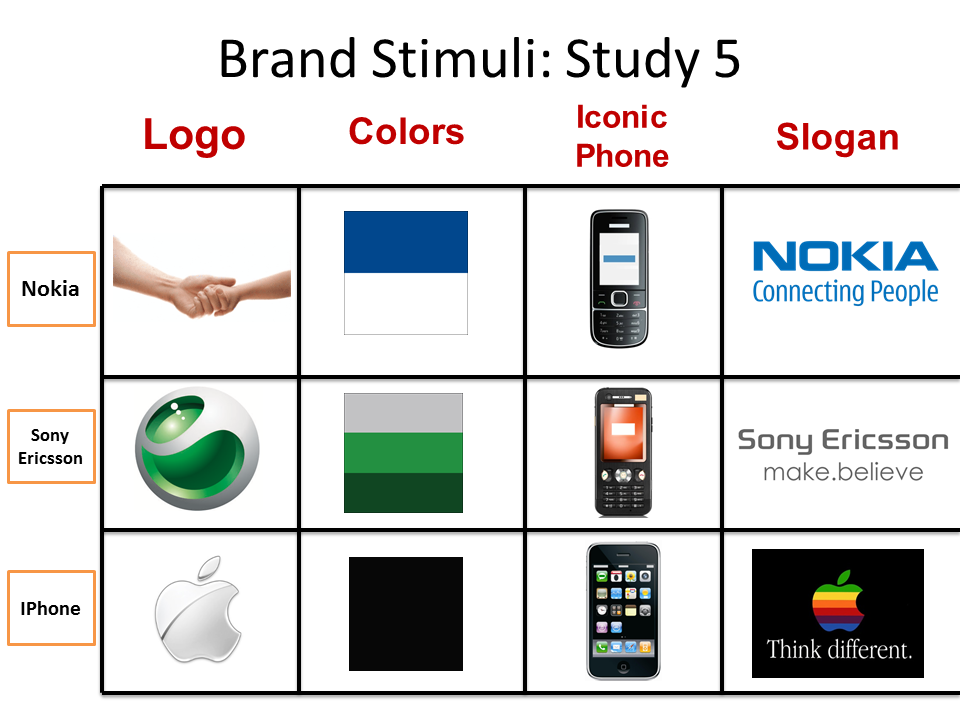

Supplement: S4 Fig — (TIF) [file pone.0121373.s004.tif]

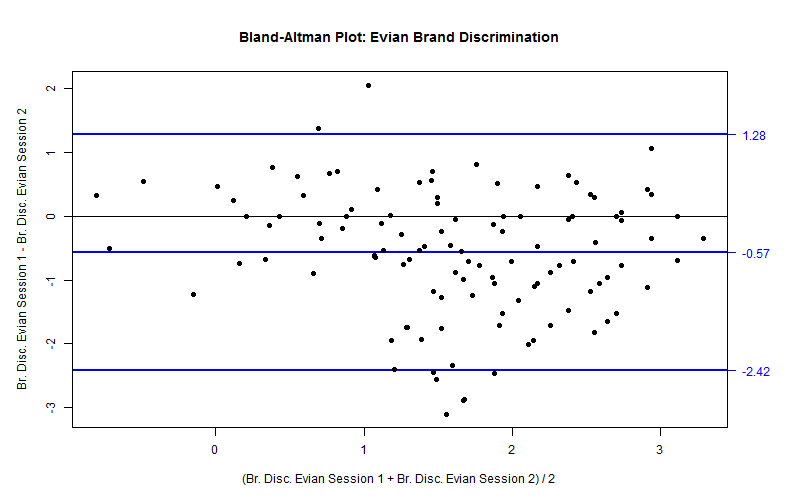

Supplement: S5 Fig — (PNG) [file pone.0121373.s005.png]

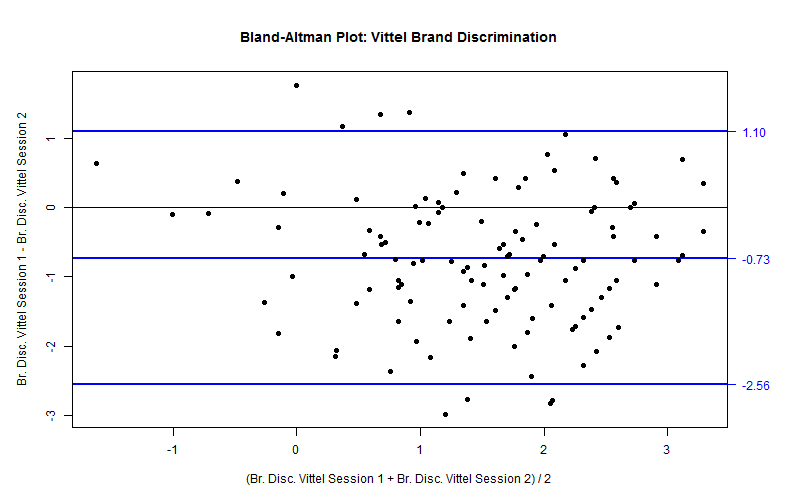

Supplement: S6 Fig — (PNG) [file pone.0121373.s006.png]
